# Contributions of RNA-seq to improve *in vitro* embryo production (IVP)

**DOI:** 10.21451/1984-3143-AR2017-0043

**Published:** 2019-10-24

**Authors:** Priscila Di Paula Bessa Santana, Artur Luis da Costa da Silva, Rommel Thiago Jucá Ramos, Arnaldo Algaranhar Gonçalves, Nathalia Nogueira da Costa, Priscilla do Carmo Azevedo Ramos, Thiago Velasco Guimarães Silva, Marcela da Silva Cordeiro, Simone do Socorro Damasceno Santos, Otávio Mitio Ohashi, Moysés dos Santos Miranda

**Affiliations:** 1 Federal Rural University of Amazon, Capitão-Poço, Pará, Brazil.; 2 Laboratory of Genomics and Bioinformatics, Federal University of Pará, Belém, PA, Brazil.; 3 Laboratory of In Vitro Fertilization, Institute of Biological Science, Federal University of Pará, Belém, PA, Brazil.

**Keywords:** biomarker genes, *in vitro* embryo production, livestock animals, RNA-seq

## Abstract

*In Vitro* Embryo Production (IVP) is widely used to improve the reproductive efficiency of livestock animals, however increasing the embryo development rates and pregnancy outcomes is still a challenge for some species. Thus, the lack of biological knowledge hinders developing specie-specific IVP protocols. Therefore, the contributions of RNA-seq to generate relevant biological knowledge and improve the efficiency of IVP in livestock animals are reviewed herein.

## Introduction

*In Vitro* Embryo Production (IVP) is a reproductive biotechnology that may be used to increase genetic gains in short generation intervals (Kasinathan *et al*., 2015), to preserve genetic diversity and to treat infertility (Comizzoli *et al*., 2010; Lima *et al*., 2013). However, improving the efficiency of IVP is a challenge in many species due to biological and technical aspects (Paramio and Izquierdo, 2014). One technical aspect is the composition of the *in vitro* culture media, and some biological aspects are the oocyte and embryo quality (Lonergan *et al*., 2006).

**Figure 1:**
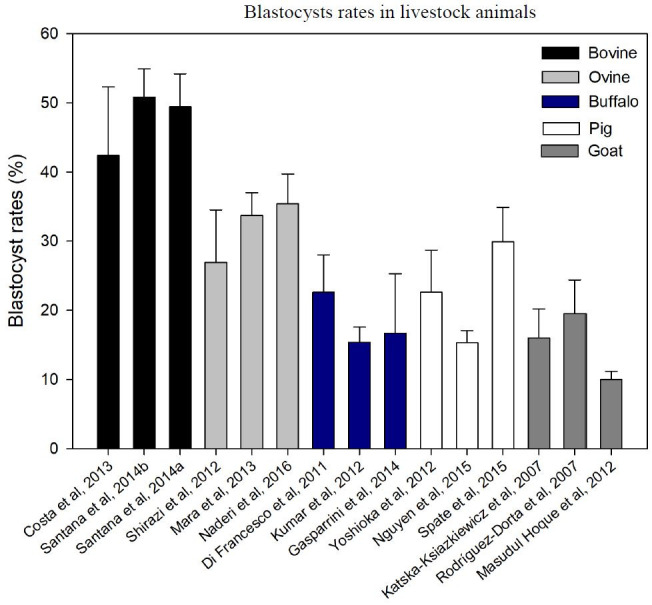
Efficiency of IVEP, measured in terms of blastocyst rates (total number of blastocysts divided by the total number of CCOs) in different livestock animals.

IVP efficiency relies on adapting the *in vitro* conditions to meet the biological requirements of each specie (De Souza-Fabjan *et al*., 2014; Grupen, 2014), which has not been done properly in many livestock animals, thus resulting in huge IVP efficiency variation. This was observed by comparing the rate of embryo development as an efficiency ratio (Fig. 1). In cattle, IVP efficiency varies from 42 to 50% (Costa *et al*., 2013; Santana *et al*., 2014a; b). However, the efficiency is lower for sheep (26-35%) (Shirazi *et al*., 2012; Mara *et al*., 2013; Naderi *et al*., 2016), buffalos (15-22%) (Di Francesco *et al*., 2011; Kumar *et al*., 2012; Gasparrini *et al*., 2014), pigs (13-22%) (Yoshioka *et al*., 2012; Nguyen *et al*., 2015; Spate *et al*., 2015), and goats (10-19%) (Kątska-Książkiewicz *et al*., 2007; Rodríguez-Dorta *et al*., 2007; Masudul Hoque *et al*., 2012). Long time research in IVP improvements certainly correlates with the high efficiency observed in cattle (Parrish, 2014), and also indicate that IVP may be potentially improved in other livestock animals if their biological requirements could be addressed.

The transcriptome of gametes and embryos has currently generated relevant biological information used to develop more suitable *in vitro* culture media for oocyte maturation and embryo culture (Prather *et al*., 2014), in addition to defining molecular markers of oocyte, sperm and embryo quality (Kropp and Khatib, 2015; Reyes *et al*., 2015; Kropp *et al*., 2017, Gilchrist *et al*., 2016) based on the transcriptomic profile of coding and non-coding RNAs. Thus, herein we discuss the current biological knowledge of gametes and embryos generated by Next Generation Sequence (NGS) technologies, and how this knowledge has been applied to improve IVP in livestock animals.

## Overview of high-throughput sequencing technologies to generate transcriptome data

The transcriptome is the whole set of transcripts in the cell and can be generated using hybridization (microarray) and Next Generation Sequencing (NGS) of RNA (RNA-seq). The main applications of transcriptome studies are to catalog mRNAs and non-coding RNAs, to infer alternative splicing events, to identify new genes, to analyse differential expression and to build coexpression networks (Wang *et al*., 2009; Han *et al*., 2015).

For microarray, the mRNA is first isolated and hybridized in a complementary and specific manner to the cDNA probes, therefore each time hybridization occurs can be identified by the fluorescence reading. Each probe is a nucleotide sequence complementary to the target gene, so it is required to know the genomic sequences of the target gene in order to build the probes; in the case the genome is not available, then the use of the microarray assay would be impaired (Pariset *et al*., 2009). This, added to the fact that the microarray slide can contain a limited number of custom probes results in a very useful technique, but with need for prior knowledge that may become a limitation depending on the specie and the type of study.

In the case of the microarray for a certain specie being unavailable, RNA-seq can be used to produce datasets of expressed sequence tag (EST) necessary to design the complementary probes, and then build a new and specie-specific microarray platform (Robert *et al*., 2011; Tsoi *et al*., 2012). Other advantages over microarray consists in RNA-seq enabling the detection of low abundant transcripts and novel genetic variants (Huang and Khatib, 2010; Dyck *et al*., 2014; Zhao *et al*., 2014), and also that RNA-seq can be used even when the genome is not available (Salvemini *et al*., 2014). In this case, assembly may be done by either the *de novo* approach or using the genome of a closely related specie as reference (Martin and Wang, 2011).

Another good point is that NGS technologies have been continuously upgraded in order to make them more affordable (Glenn, 2011). As a result, there are many NGS platforms available that differ in several technical aspects (method of library preparation, chemistry and sequencing efficiencies), but share one feature in generating thousands of reads (Liu *et al*., 2012). Affordable cost and high efficiency may be related to the increasing number of sequenced genomes available in online Databases (see www.ncbi.nlm.nih.gov/genbank/statistics), as well as the number of transcriptomes in public repositories such as GEO (Gene Expression Omnibus, www.ncbi.nlm.nih.gov/ geo) and SRA (Sequence Read Archive, www.ncbi.nlm.nih.gov/sra).

The amount of RNA-seq studies of livestock animals aimed at reproduction biotechnology aspects has also increased over the years. RNA-seq has been predominantly performed with SOLiD™, Ion™ and Illumina™ platforms, mainly to compare the transcriptome profile of mRNAs and miRNAs in different cell types and conditions (differential expression analysis). In the following sections we discuss some RNA-seq applications with differential expression analysis such as the study of non-invasive biomarkers of oocyte competence, sperm and embryo quality, the onset of Embryo Genome Activation (EGA), study of oocyte maturation and embryo development, as well as the effects of *In Vitro* Fertilization (IVF) on these processes (Tab.1).

**Table 1:** Aplications and brief summary of RNA-seq studies in livestock animals.

Applications of RNA-seq	Reference	Specie	RNA-seq plataform	Brief summary of experimental design
Produce dataset of EST to design probes and a new microarray platform	Robert *et al*. (2011)	Bovine	Genome Sequencer FLX, 454 Life Sciences	Sequencing of germinal vesicle oocyte, 2-, 4-, 8-, 16-cells, morula, blastocyst and hatched blastocyst produced *in vitro* and derived *in vivo*
	Tsoi *et al*. (2012)	Porcine	Genome Sequencer FLX, 454 Life Sciences	Sequencing of germinal vesicle, metaphase II oocyte, 2-, 4-, 8-, morula, expanded and hatched blastocyst produced *in vitro* and derived *in vivo*
Study oocyte competence	Macaulay *et al*. (2014)	Bovine	Illumina, HiSeq 2000	Comparison of germinal vesicle *versus* metaphase II oocytes matured *in vitro*
	Reyes *et al*. (2015)	Bovine	Illumina, HiSeq 2000	Comparison of polyadenylated transcripts in germinal vesicle *versus* metaphase II oocytes matured *in vitro*
	Macaulay *et al*. (2016)	Bovine	Illumina, HiSeq 2000	Comparison of germinal vesicle *versus* metaphase II oocytes *in vitro* matured *versus* total RNA found in the TZPs
	Gilchrist *et al*. (2016)	Bovine	Illumina, HiSeq 2500	Comparison of germinal vesicle *versus* metaphase II oocytes *versus* presumptive zygotes
Study non-invasive **mRNA** and **miRNA** biomarkers of oocyte competence	Luo *et al*. (2016)	Sheep	Illumina, HiSeq 2000	Comparison of granulosa cells isolated from animals short-term dietary-restricted *versus* nutrient-supplemented
	Wu *et al*. (2017)	Sheep	Illumina, HiSeq 2500	Comparison of granulosa cells isolated from prepubertal *versus* adult superstimulated follicles
	Mazzoni *et al*. (2017)	Bovine	Illumina, HiSeq 2500	Sequencing of granulosa cells collected at OPU prior to IVEP. Follow IVEP and correlation of the blastocyst rates with RNA-seq results
Study transcriptomic profile and molecular markers of sperm quality	Card *et al*. (2013)	Bovine	Illumina, HiSeq 2000	Sequencing of cryopreserved spermatozoa
	Kropp *et al*. (2017)	Bovine	Illumina, HiSeq 2000	Comparison of blastocysts derived from low-fertility *versus* high-fertility bull (based on SCR)
	Selvaraju *et al*. (2017)	Bovine	Ion ProtonTM and Illumina, HiSeq 2000	Use of two NGS platafforms to sequence spermatozoa cells obtained from fresh semen
	Gòdia *et al*. (2018)	Porcine	Illumina, HiSeq 2000	Optimization of methodological workflow for RNA-seq of spermatozoa from fresh semen
Study the onset of EGA	Ostrup *et al*. (2013)	Porcine	SOLiD, Applied Biosystems	Comparison of 2- and 4-cells embryos *in vitro* produced *versus in vivo* derived
	Graf *et al*. (2014)	Bovine	Illumina, GAIIx	Comparison of germinal vesicle oocyte, metaphase II oocyte, 4-, 8-, 16-cells and blastocysts produced *in vitro*
	Jiang *et al*. (2014)	Bovine	SOLiD, Applied Biosystems	Comparison of metaphase II oocyte, 2-, 4-, 8-, 16-cells, early morula, late morula and blastocyst derived *in vivo*
	Jiang *et al*. (2015)	Bovine, human, mice and pig	RNA-seq data downloaded from online database	Comparison of metaphase II oocyte, 2-, 4-, 8-, 16-cells, early morula, late morula and blastocyst derived *in vivo*
Study the IVF effects on embryo development	Bauer *et al*. (2010)	Porcine	Illumina, GAIIx	Comparison of blastocysts *in vivo* derived *versus* collected from uterus at 2-4-cells further *in vitro* cultured until blastocyst stage
	Huang and Khatib (2010)	Bovine	Illumina, GAIIx	Comparison of fully developed *in vitro* blastocyst *versus* arrested embryos (failed to develop from morula to blastocyst stage)
	Redel *et al*. (2012)	Porcine	Illumina, GAIIx	Comparison of embryos *in vitro* cultured in low or high O2 atmospheres
	Driver *et al*. (2012)	Bovine	Illumina, Hiseq 2000	Comparison of *in vitro* and *in vivo* blastocysts with similar quality grade
	Cao *et al*. (2014)	Porcine	SOLiD 4, Applied Biosystems	Comparison of 1-, 2-, 4-, 8-cells, morula and blastocysts *in vivo* derived *versus* produced *in vitro* using SCNT biotechnology
	Hera *et al.* (2016)	Bovine	Illumina, HiSeq 2500	Comparison of blastocysts *in vitro* produced in serum-containing media *versus* serum free-media *versus* blastocysts derived *in vivo*
	Milazzoto *et al.* (2016)	Bovine	Illumina, HiSeq 2000	Comparison of slow and fast-cleavage embryos *in vitro* produced *versus* embryos derived *in vivo*
Study non-invasive **mRNA** and **miRNA** biomarkers of embryo quality	Kropp *et al*. (2014)	Bovine	Illumina, GAIIx	Comparison of media conditioned by fully developed blastocysts *versus* arrested embryos (failed to develop from morula to blastocyst)
	Kropp and Khatib (2015)	Bovine	Illumina, GAIIx	Comparison of media conditioned by fully developed blastocysts versus arrested embryos (failed to develop from morula to blastocyst)

## Transcriptomic profile of gametes and embryos

Next, how RNA-seq studies have been applied to describe novel remarks in reproductive biology issues are reviewed, such as the genetic mechanisms of oocyte maturation, development and metabolism of pre-implantation embryos, and the effects of *in vitro* conditions on gametes and embryos. Furthermore, the use of RNA-seq to identify molecular markers of oocyte, sperm and embryo quality are reviewed in the following sections and are summarized in Table 2. Lastly, how this knowledge has been used to improve IVP is presented.

**Table 2:** Summary of putative biomarkers reported by RNA-seq for selection of good quality gametes and embryos.

Source	Putative biomarker genes
MII oocyte	*CCNB1, WEE2, FBXO43, MELK, H1FOO, RALB, ZP2, IGF2R,* miRNA-155, miRNA-222, miRNA-21 and miRNA-190a
Transzonal projections	*ZNF773*, *ZNF689*, *ZNF75A*, *ZNF664*, *ZNF395, FAM21* and *WASF2*
*Cumulus* cells	*GATM*, *Mx1* and *STC1*
Spermatozoa	*CLU, AKAP4, PRM1, PRM2, PLCZ1, CRISP2, PSG-1, HLA-E, DBY, EIF1, EIF5, EEF1A1, EEF1γ, TFB2M* and *CYCS*
Blastocyst	*DNMT3A, GATA3, CD9, ATP6V0A4, FAM115C, LGALS1, SLC9A3R1, BCAM, BPIFA1, PLXNA3, SHROOM2, SLC16A7, EEF2, RPL10A* and *RPL38*
IVC-conditioned media	*POSTN, VSNL-1, PUM2,* miRNA-181a2, miRNA-196a2, miRNA-302c, and miRNA-25

## Blastocysts rates in livestock animals

## Understanding the molecular aspects of oocyte competence and maturation

Molecular aspects of oocyte competence deeply influence embryo development and IVP outcomes (Yuan *et al*., 2017). Hence, improving oocyte competence becomes a major challenge in IVP (Gilchrist and Thompson, 2007). What do we know about the cytoplasmic components that determine an oocyte’s developmental potential? RNA-seq studies have been used to uncover novel markers associated with oocyte quality.

During oogenesis in the germinal vesicle stage (GV), mammalian oocytes store several transcripts that support the development of early embryos. Afterwards, the germinal vesicle breakdown occurs during oocyte maturation and the transcriptional activity becomes silenced. At the same time, the overall RNA content decreases in the metaphase II oocytes (MII) due to mRNA translation and degradation processes (reviewed by Labrecque and Sirard, 2014).

Indeed, evidence from a RNA-seq study in bovines has confirmed that the overall RNA content decreased from GV (10,181) to MII (8,941) stages. Also, the cytoplasmic polyadenylation of the mRNA content which indicates the occurrence of translation during oocyte maturation has been demonstrated. The study identified 2,455 (23%) differentially expressed genes (DEG) between immature and mature *in vitro* oocytes, for which 503 (20%) transcripts were up-regulated in MII oocytes. Most of the up-regulated transcripts were involved in cell-cycle progression (*CCNB1*, *WEE2*, *FBXO43*, and *MELK*), which were also polyadenylated indicating their translation. Therefore this suggests that part of the mRNA stored by oocytes goes to translation during *in vitro* maturation, probably to support meiosis resumption (Reyes *et al*., 2015).

However, if an oocyte becomes transcriptionally silent after germinal vesicle breakdown, how can the presence of up-regulated transcripts in MII oocytes be explained? Regarding this, a RNA-seq study in cattle reported that *cumulus* cells produce transcripts which can be transferred to the oocyte. Using confocal live cell imaging, the newly synthetized RNA label with [3H]-uridine was detected along the length of the transzonal projections, confirming the *cumulus* cells as a novel source of *de novo* transcripts to the oocyte (Macaulay *et al*., 2014). The RNA-seq study of the transzonal projections content identified 624 transcripts corresponding to long non-coding RNAs, uncharacterized transcripts and known mRNAs such as zinc fingers (*ZNF773*, *ZNF689*, *ZNF75A*, *ZNF664*, and *ZNF395*), which regulate transcription by DNA binding, and transcripts (*FAM21* and *WASF2*) for actin- and cytoskeleton-related process (Macaulay *et al*., 2016).

To this point, some transcripts involved in modulating the cell cycle and transcription produced by either oocytes or *cumulus* cells seem to be important for the maturation process, and thus may be correlated with oocyte competence. The transcriptome profile of miRNAs in GV, MII (*in vitro* mature) and early zygotes (Gilchrist *et al*., 2016) in cattle also reinforces this correlation. The most abundant miRNAs during oocyte maturation were linked to target genes involved in transcription regulation by RNA polymerase II activity. Some miRNAs were up-regulated between MII and early zygote stages (miRNA-155, -222, and -21), while others were down-regulated (miRNA-190a). Considering that miRNAs mostly regulate gene expression through translation repression and mRNA degradation, any increase in its levels causes a decrease in the performance of the target transcripts (Ambros, 2001). Therefore, MII oocytes have miRNA to both increase and decrease the mRNA levels in early zygotes, which may be used as molecular biomarkers of oocyte competence if their roles in embryo development are clarified.

In summary, the transcriptome profile of mRNAs and miRNAs during maturation (Reyes *et al*., 2015; Gilchrist *et al*., 2016; Macaulay *et al*., 2016) all suggest the role of transcripts regulating the cell cycle and transcription as potential markers of molecular quality in oocytes.

## Selection of non-invasive biomarkers of oocyte competence

The importance of the granulosa cells are widely known as being beneficial for IVP, such as in the removal of *cumulus* cells from *Cumulus*-Oocyte Complexes (COCs) which significantly decreases the maturation rate (35.4%) compared to intact COCs (79.5%), (Macaulay *et al*., 2016). Given their close relation with oocyte quality, the transcriptome profile of*cumulus* has been performed in cows (Bunel *et al*., 2015), sheep (Luo *et al*., 2016; Wu *et al*., 2017) and humans (Feuerstein *et al*., 2012) as a non-invasive strategy for evaluating the quality of oocytes.

The non-invasive methods for oocyte selection might be particularly useful for OPU (Ovum Pick-Up) -IVP protocols. This is because based on the granulosa transcriptome analysis only the high-potential COCs prior to *in vitro* fertilization may be selected, thus increasing the embryo development rates. In cows, a microarray study compared the overall gene expression patterns in biopsies of *cumulus* cells collected from COCs, which were further evaluated for embryo development. The expression pattern in biopsies from COCs that developed into blastocysts was different from the COCs which failed (68 DEG). *GATM,* involved in amino acid metabolism and free radical scavenging, was the highly expressed gene in the blastocyst fate (Bunel *et al*., 2015) and might be useful as a biomarker to select high-potential COCs.

The second advantage for IVP is based on the transcriptome profile of the follicular cells making it possible to select the oocyte donors with the best quality COCs for OPU-IVP. This is due to the RNA-seq study reporting a high correlation between the gene expression patterns of follicular cells and embryo development. Regarding this, COCs from 24 Holstein cows were separately *in vitro* fertilized for their embryo development rates to be correlated with the profile of the respective follicular cells. As a result, 2 genes were positively associated (*Mx1* and *STC1*), which means that all the cows with higher blastocyst rates also presented both genes as up-regulated. *STC1* (Stanniocalcin-1) was particularly postulated as a potential biomarker of oocyte quality due to its role in granulosa cell development (Mazzoni *et al*., 2017).

## Selecting biomarkers of sperm quality

Spermatozoa potentially contribute to embryo development by delivering DNA, a centriole, transcription factors, signaling molecules and a variety of coding and non-coding RNAs during fertilization (reviewed by Krawetz *et al.*, 2005). RNA-seq studies of spermatozoa have been useful to identify novel markers and verify known markers of sperm quality, as well as to investigate the correlation between sperm quality and embryo development.

The first RNA-seq in spermatozoa was reported by Card *et al*. (2013) in cattle, and confirmed many mRNAs found by previous microarrays such as *CLU*, *AKAP4*, *PRM1, PRM2, PLCZ1*, *CRISP2, PSG-1, HLA-E* and *DBY*. The authors found 6,166 transcripts, in which 368 (~6%) were considered full-length transcripts (FPKM>100), and in addition they identified transcripts that had not been reported in the previous microarrays such as translation initiation (*EIF1* and *EIF5*) and elongation (*EEF1A1* and *EEF1γ*) factors. Among the above transcripts, *CLU* (clusterin), *PLCZ1* (phospholipase Cζ) and *PRM1* (protamine-1) have been reported as potential molecular markers of sperm quality (Selvaraju *et al*., 2017); their delivery by sperm into the oocyte upon fertilization also suggests their roles in embryo development (Krawetz *et al*., 2005). Gòdia *et al*. (2018) recently reported the first RNA-seq study of boar sperm. However, no conclusive evidence was generated since the methodology is still being optimized for spermatozoa purification and library preparation.

Using biomarkers may be useful for selecting high fertility bulls in IVP programs. An RNA-seq study reported a correlation between the transcriptomic profile of blastocysts and bull fertility measured by sire conception rate (Kropp *et al*., 2017). Despite similar morphology, gene expression patterns of embryos derived from high and low fertility sirings were different (98 DEG genes). Next, bisulfite sequencing of sperm from high and low fertility sires revealed 76 differentially methylated regions, suggesting that methylation of sperm chromatin influences the gene expression patterns of pre-implantation embryos. The genes associated with mitochondrial and cytochrome C functions, such as *TFB2*M and *CYCS* respectively, were upregulated in embryos fertilized with high sire conception rate bulls. In addition, *TFB2*M mRNA silencing significantly reduced the blastocyst rate, indicating that its expression by the derived embryos may be used as a potential biomarker to select high fertility bulls (Kropp *et al*., 2017). In summary, the use of RNA-seq to identify novel molecular markers of sperm quality and correlate them with embryo development may help to improve IVP outcomes by selecting highly fertile bulls.

## Understanding the genetic mechanisms of embryo development

During *in vitro* culture (IVC) important genetic events such as the maternal-zygotic transition (MZT) and the small and large waves of embryo genome activation (EGA) take place. In fact, the maternal genome controls all aspects of embryo development until EGA through mRNA and proteins stored in the oocyte cytoplasm. MZT occurs as the embryo develops; first, the maternal mRNA is gradually eliminated, and then zygotic transcription is initiated (reviewed by Tadros and Lipshitz, 2009). Understanding MZT and EGA processes is particularly useful for IVP, since the transcriptome profile of embryos as well as the metabolic requirements completely change with their occurrence. Thus, knowing the onset of EGA is important when trying to establish an IVP protocol, as EGA timing varies among species.

Experiments with α-amanitin and the incorporation of [3H] uridine helped to define the onset of EGA at 8 to 16 cells in cattle and sheep (Telford *et al*., 1990), at 2 to 4 cells in buffalos (Kumar *et al*., 2012), and at the 4 to 8-cell stage in pigs (Memili and First, 1998). The RNA-seq approach has introduced new strategies to identify the onset of EGA. The first approach was to detect DEG genes between embryos and oocytes. The second approach was to detect transcripts from the paternal allele using the identification of specific SNPs. Finally, the third approach was to detect incompletely processed transcripts identified by the presence of intronic sequences due to incomplete co-transcriptional splicing (Graf *et al*., 2014).

Novel strategies of RNA-seq have helped to determine that the onset of EGA occurred more prematurely than previously thought in cows. It was observed that the small EGA wave occurs at 4 to 8 cells, and the large EGA wave at 8 to 16 cells (Jiang *et al*., 2014). In this study, the authors compared the transcriptome profile of oocytes in metaphase II, embryos with 2, 4, 8, and 16 cells, morula and blastocyst produced *in vivo*. About 2,845 DEG genes between all stages of development were described, in which 2,031 genes were just between the 4 to 8-cell stages, confirming the onset of minor EGA in this period.

A RNA-seq study in pigs also helped to define that EGA occurs earlier than previously thought, which is at the 2 to 4-cell stage with 2,101 DEG genes between the stages. Transcripts related to protein synthesis were predominantly found in 2-cell embryos, indicating the translation of transcripts inherited from the oocyte. This was in contrast to the 4-cell embryos which showed transcripts for “RNA splicing and processing” and “DNA metabolic processes” in agreement with the start of transcription activity by zygotes, which is an EGA hallmark (Østrup *et al*., 2013).

Data availability in *online* databases currently provides the chance to compare the transcriptome profiles of embryos from different species, thereby being able to find similarities in their embryo development programs (Jiang *et al*., 2015). For this, RNA-seq data was used to perform a co-expression network analysis with all the embryo development stages from bovine, human and mouse species. Surprisingly, the comparison between the human and bovine transcriptome profiles showed more similarities than those of human and mouse, suggesting that bovine embryos are better models for human embryonic development than mouse embryos (Jiang *et al*., 2014).

## New insights about the metabolism of pre-implantation embryos

Embryo metabolism is an important issue, especially during the IVC step. In general, they have been managed like any somatic cell *in vitro* culture, while a fundamental question has remained unsolved: if embryos can divide as quickly as cancer cells, is their glucose metabolism also similar? The favorable speculations in the literature (Vander Heiden *et al*., 2009; Krisher and Prather, 2012) were suddenly confirmed by a series of transcriptomic studies.

A RNA-seq study of *in vitro* blastocysts in pigs reported the expression of gene variants of hexokinase (*HK*) and pyruvate kinase (*PKM2*) related to slowing the tricarboxylic acid cycle and increasing the pentose phosphate pathway; a behavior compatible to the Warburg effect (Redel *et al*., 2012). A RNA-seq study of *in vivo* produced embryos in cows also reported the metabolic behavior of the Warburg effect. Based on a comparison of the transcriptomic profiles of oocytes and different stages of pre-implantation embryos, a range of 11,488 to 12,729 genes including several metabolic pathways such as the pentoses-phosphate, glycolysis, oxidative phosphorylation, and the tricarboxylic acid cycle were identified (Jiang *et al*., 2014).

The Warburg effect was proposed to explain the metabolic behavior of cancer cells that mostly depend on aerobic glycolysis instead of mitochondrial oxidative phosphorylation. The behavior consequently facilitates the uptake and incorporation of nutrients into the biomass (nucleotides, amino acids and lipids) which becomes the fuel needed to produce new cells (Vander Heiden *et al*., 2009) and to trigger rapid cell proliferation. Hence, this consists in an advantageous metabolic adaptation to the cancer cells, and also to the developing embryo. While glucose is shifted to the Warburg effect, the hypothesis is that the embryo depends on the β-oxidation of the fatty acids to provide the required ATP for the cells (Krisher and Prather, 2012).

New insights about the metabolism of carbohydrates and fatty acids of pre-implantational embryos have encouraged studies of novel supplements in IVC media able to induce the Warburg effect in embryos. The use of PS48 (a PDK1 activator) in pigs increased blastocyst formation and the total number of cells, possibly by increasing the phosphorylation of protein kinase B (*PKB/Akt*), which activates the Warburg effect (Spate *et al*., 2015). Similarly, arginine added to the IVC media increased blastocyst rate and the total number of cells in pigs (Redel *et al*., 2015), and also blastocyst quality and hatching in bovines (Santana *et al*., 2014a). The putative mechanism occurred through *mTOR* phosphorylation (mammalian target of the rapamycin complex), which also activates the Warburg effect (Redel *et al*., 2015). Thus, the use of supplements in IVC media for activating the Warburg effect have been shown to improve *in vitro* embryo development. That strategy could never be tested if the previous RNA-seq studies had not evidenced the Warburg metabolic behavior in embryos from different livestock animals.

## Understanding the *in vitro* culture influence on embryo quality

IVC is a critical step of IVP due to specific chemical and physical conditions that allow embryo development from the zygote to the blastocyst stage. Regarding this, the intrauterine and oviduct microenvironment is considered as the “gold standard” model for IVC (Tervit *et al*., 1972) because its composition quickly changes in response to physiological regulation in order to provide the substances required by each stage of embryo development (Knobil *et al*., 2006). Given the multiplicity of embryonic development and the fluid complexity, it is reasonable to think that IVC conditions are not entirely appropriate and affect embryo quality.

It is known that morphological and molecular differences between *in vitro* and *in vivo* embryos are directly associated with sub-optimal *in vitro* culture conditions (Niemann and Wrenzycki, 2000; Lonergan *et al*., 2003; Lonergan *et al*., 2006; Rizos *et al*., 2008), and this has also been confirmed by high throughput technologies. A RNA-seq study in cattle compared the transcriptomic profile of *in vitro* and *in vivo* derived blastocysts which presented good quality according to International Embryo Transfer Society (IETS) standards (Stringfellow and Seidel, 1998), and as a result found 793 DEG genes (Driver *et al*., 2012). This result was considerably larger than a previous microarray that identified the expression of 384 genes, of which 85% (326) were differentially expressed between the conditions (Corcoran *et al*., 2006).

One strategy to improve the *in vitro* culture media is testing supplementations in media followed by the gene expression analysis of the *in vitro* produced embryo compared to the *in vivo* counterpart (Lonergan *et al*., 2006; VanGuilder *et al*., 2008). Fetal Bovine Serum (FBS) is one that is worthy of investigation. A RNA-seq study compared the transcriptome profile of bovine embryos produced in either FBS-containing or FBS-free media to the profile of *in vivo* blastocysts to address the effects of FBS on embryo quality (Hera *et al*., 2016). Surprisingly, FBS-free embryos were more similar to *in vivo* embryos, having five times fewer DEG genes (207) than FBS-containing embryos (1,109). However, after looking at the lipid and amino acid pathway gene expression patterns, it was concluded that FBS-free and *in vivo*-derived embryos were still very different. This may be related to the role that high concentrations of FBS cause an accumulation of lipids and decrease cryotolerance in *in vitro* embryos (Abe *et al*., 2002).

It is known that media can affect embryo quality, and some evidence also suggests that embryos can modify their own expression in response to the media. According to RNA-seq studies, bovine embryos produced *in vitro* in FBS-free media showed over-expression of the cholesterol biosynthesis pathway in comparison to the *in vivo* derived embryos (Driver *et al*., 2012; Hera *et al*., 2016). Thus, it was speculated that there would be a mechanism to compensate the deficit of lipids in an *in vitro* system, where they are needed to produce membrane phospholipids and metabolic energy. This also may be related to the correlation of FBS supplementation and increased rates of blastocyst formation in *in vitro* culture systems (George *et al*., 2008). Taken together, embryos can modulate their lipid metabolism in response to the media; however, there is no consensus if it is beneficial to the *in vitro* embryo development.

## Selection of non-invasive biomarkers of embryo quality and pregnancy success

The “gold standard” of embryo quality is successful implantation and pregnancy (Van Soom *et al*., 2003). But how can it be possible to know which embryo is capable of implanting and result in pregnancy before the transfer? Surprisingly, evidence from transcriptomes has shown that good-quality embryos can signal their developmental competence, so the endometrium positively selects them for establishing pregnancy (Macklon and Brosens, 2014). This so-called “selective notion” by the endometrium is reasonable, since successful implantation depends on complex interactions between embryo and endometrial cells through hormonal regulation and cross-talk of several molecular signals (reviewed by Bazer *et al*., 2010).

The first evidence of the endometrium “selective notion” was a microarray study of endometrial cells obtained from pregnant cows (Mansouri-Attia *et al*., 2009). The transcriptomic profiles of endometrial cells after the transfer of embryos produced by IVF, Artificial Insemination (AI), and Nuclear Somatic Cell Transfer (TNCS) were significantly different based on the analysis of DEG genes, suggesting that embryo quality can influence the response of the endometrium for establishing pregnancy. In humans, the different transcriptomic profile of endometrial cells exposed to media conditioned by good quality (15 DEG genes) and by non-viable embryos (449 DEG genes) also raised the correlation between embryo quality and the endometrium response (Brosens *et al*., 2014).

If the endometrium can select embryos, the next question is: how do embryos signal their development potential? Notably, it was shown that a serine protease released by mouse embryos (trypsin) elicits cascade effects ending up with the release of prostaglandin E2 in endometrial epithelial cells, which in turn led to decidualization and implantation (Ruan *et al*., 2012).

RNA-seq studies in cows identified mRNAs and miRNAs that possibly act as signal molecules, although this requires further investigation. Regarding this, 11 miRNAs (Kropp *et al*., 2014) and 17 mRNAs (Kropp and Khatib, 2015) were found differentially expressed in culture media samples, conditioned by the culture of arrested *versus* fully-developed blastocysts. Among the mRNAs secreted by embryos, at least one had been previously reported (*POSTN*). Ovine endometrial cells can also produce *POSTN* transcripts and protein, which may stimulate the attachment of trophectoderm cells *in vitro* (Ahn *et al*., 2009). Taken together, these findings raise the idea that embryos can release more than one type of signal molecule to induce endometrial response. Identifying the embryo signals and understanding how to induce their production and release will certainly open new perspectives in reproductive biology, particularly for IVP.

POSTN mRNA encodes a protein that binds to integrins to support adhesion and migration of epithelial cells, and VSNL-1 mRNA encodes a protein that modulates intracellular signaling pathways regulating the activity of adenylyl cyclase; both were found highly induced in IVC conditioned media. Hence, the inhibition of POSTN translation resulted in a significant decrease of the blastocyst rate in cows (Kropp and Khatib, 2015), confirming its pivotal role in embryo development, and also as a candidate for being a molecular marker of embryo quality. Based on this evidence, the quantification of *POSTN* and *VSNL-1* mRNA levels in IVC media may be a potential non-invasive strategy to select good quality embryos, although further studies about pregnancy and calving outcomes may clarify if they are also eligible for transfer in IVP programs.

In brief, RNA-seq data has been useful to investigate molecular biomarkers of embryo quality and non-invasive strategies to select good quality embryos for transfer; taken together, these results can help select embryos that are theoretically most capable for implantation. In the long term, this may increase pregnancy rates and the efficiency of IVP in many livestock animals.

## Future Perspectives

The use of biomarker genes to select competent oocytes in IVP protocols may increase the rates of blastocyst development, and help to select the best donor cows for OPU-IVP. In parallel, TNCS and transgenesis biotechnologies can also take advantage of the oocyte selection strategy to improve the blastocyst rates. Similarly, the use of biomarker genes to select good quality embryos may help to increase the outcome of IVP in terms of pregnancy rates.

Following the reports of biomarker genes, the future challenge will be correlations with embryo development and pregnancy rates. With this aim, it may be useful to use techniques to inhibit mRNA translation and verify the effect of gene function loss on embryo development (Betts *et al*., 2014), as well as supplement *in vitro* culture media with substances able to inhibit and induce their expression levels. Further, proteomic and metabolomic studies may help to clarify the post-transcriptional and translational regulation mechanisms.
